# Rationale, Design, and Baseline Characteristics of Beijing Prediabetes Reversion Program: A Randomized Controlled Clinical Trial to Evaluate the Efficacy of Lifestyle Intervention and/or Pioglitazone in Reversion to Normal Glucose Tolerance in Prediabetes

**DOI:** 10.1155/2017/7602408

**Published:** 2017-01-12

**Authors:** Yingying Luo, Sanjoy K. Paul, Xianghai Zhou, Cuiqing Chang, Wei Chen, Xiaohui Guo, Jinkui Yang, Linong Ji, Hongyuan Wang

**Affiliations:** ^1^Department of Endocrinology and Metabolism, Peking University People's Hospital, Beijing, China; ^2^Melbourne EpiCentre, University of Melbourne, Melbourne, VIC, Australia; ^3^Clinical Trials and Biostatistics Unit, QIMR Berghofer Medical Research Institute, Brisbane, QLD, Australia; ^4^Institute of Sports Medicine, Peking University Third Hospital, Beijing, China; ^5^Department of Parenteral and Enteral Nutrition, Peking Union Medical College Hospital, Beijing, China; ^6^Department of Endocrinology and Metabolism, Peking University First Hospital, Beijing, China; ^7^Department of Endocrinology and Metabolism, Beijing Tongren Hospital, Capital Medical University, Beijing, China; ^8^Department of Epidemiology and Biostatistics, School of Public Health, Peking University Health Science Center, Beijing, China

## Abstract

*Background*. Patients with prediabetes are at high risk for diabetes and cardiovascular disease (CVD). No study has explored whether intervention could revert prediabetes to normal glycemic status as the primary outcome. Beijing Prediabetes Reversion Program (BPRP) would evaluate whether intensive lifestyle modification and/or pioglitazone could revert prediabetic state to normoglycemia and improve the risk factors of CVD as well.* Methods*. BPRP is a randomized, multicenter, 2 × 2 factorial design study. Participants diagnosed as prediabetes were randomized into four groups (conventional/intensive lifestyle intervention and 30 mg pioglitazone/placebo) with a three-year follow-up. The primary endpoint was conversion into normal glucose tolerance. The trial would recruit 2000 participants (500 in each arm).* Results*. Between March 2007 and March 2011, 1945 participants were randomized. At baseline, the individuals were 53 ± 10 years old, with median BMI 26.0 (23.9, 28.2) kg/m^2^ and HbA1c 5.8 (5.6, 6.1)%. 85% of the participants had IGT and 15% had IFG. Parameters relevant to glucose, lipids, blood pressure, lifestyle, and other metabolic markers were similar between conventional and intensive lifestyle intervention group at baseline.* Conclusion*. BPRP was the first study to determine if lifestyle modification and/or pioglitazone could revert prediabetic state to normoglycemia in Chinese population. Major baseline parameters were balanced between two lifestyle intervention groups. This trial is registered with www.chictr.org.cn: ChiCTR-PRC-06000005.

## 1. Introduction

About 6.9% of adults are estimated to have IGT (impaired glucose tolerance) globally, and the IGT prevalence is likely to be increased to 8% by 2035 [1]. The burden of prediabetes is significantly high in China. The national survey conducted during 2007-08 reported 15.5% prevalence of prediabetes in adult Chinese population [2]. Patients with prediabetes are at high risk for both diabetes and its complications, especially the cardiovascular disease (CVD) [3, 4]. Studies have shown that 1.5%–7.4% of individuals with prediabetes develop type 2 diabetes annually [4]. After 3–5 years of follow-up, 1/4 of the patients with prediabetes would develop type 2 diabetes [4]. The rate of incidence of diabetes in the control arm of Da Qing study was 15.7 per 1000 person years, during 6 years of follow-up [5]. Patients with prediabetes were also at high risk of developing CVD [6].

Over the last two decades, studies in China and other countries had shown that lifestyle intervention, with or without therapeutic intervention, could decrease the risk of developing type 2 diabetes in patients with prediabetes [5, 7–13]. In Da Qing study, after 6 years of follow-up, compared with control group, relative risk of developing type 2 diabetes was reduced by 42% in intensive lifestyle intervention group [5]. Finnish diabetes prevention study (DPS) compared the efficacies of lifestyle intervention in preventing diabetes in patients with prediabetes [8]. The incidence of diabetes in intervention group was less than half of that in control group after two years of follow-up. Diabetes prevention program (DPP) also showed that intensive lifestyle intervention in patients with prediabetes could prevent the development of type 2 diabetes in 1 of 7 followed up over 3 years [7]. Apart from lifestyle intervention, use of antidiabetes drugs (ADDs) had shown the effectiveness in preventing diabetes in patients with prediabetes [7, 10–14]. As compared with placebo, medication intervention might reduce the relative risk of developing diabetes by 25–60% and might increase the possibility of conversion rate up to 70% [10, 11, 14–16].

Besides the prevention of diabetes, lifestyle intervention may also decrease the risk of CVD and mortality in prediabetes population. The 23-year follow-up data from the Da Qing study showed that the cumulative incidence of cardiovascular disease mortality was decreased from 19.6% to 11.9% in the lifestyle intervention group [17]. All-cause mortality was also decreased from 38.4% to 28.1% after an initial 6 years of lifestyle intervention [17]. Some studies also suggested that patients with prediabetes might also get some potential cardiovascular benefits from antidiabetic drugs since many surrogate markers were improved [18, 19].

Until now, three thiazolidinedione (TZD) drugs, including troglitazone, were used to prevent diabetes in IGT population. Troglitazone markedly reduced the incidence of diabetes during its limited period of use compared with all the other interventions in the DPP study [16]. Later on, rosiglitazone and pioglitazone were also tested to prevent diabetes in IGT population. Both of them showed a significant effect on reducing the risk of developing diabetes [4, 11, 12, 17]. However, no study have explored whether intervention could revert prediabetes to normal glycemic status as the primary outcome. The earlier studies were aimed at evaluating the efficacy of intervention on preventing progression to diabetes in individuals with impaired glucose tolerance (IGT), not prediabetes.

The purpose of Beijing Prediabetes Reversion Program (BPRP) was to examine whether lifestyle modification with or without pioglitazone could revert prediabetic state to normoglycemia over 3 years of follow-up in patients with prediabetes. Apart from the description of the study protocol of BPRP, the baseline characteristics of the randomized study subjects, by age groups and lifestyle intervention status, are also presented in this study.

## 2. Materials and Method

### 2.1. Study Design

Beijing Prediabetes Reversion Program (BPRP) is a prospective, multicenter, randomized, double blinded, and placebo controlled clinical trial, based on a 2 × 2 factorial design. Patients with prediabetes were randomized into four groups: conventional lifestyle intervention + placebo, conventional lifestyle intervention + pioglitazone hydrochloride 30 mg daily, intensive lifestyle intervention + placebo, and intensive lifestyle intervention + pioglitazone hydrochloride 30 mg daily. The study hypothesis was that intensive lifestyle intervention and/or pioglitazone 30 mg QD would increase the conversion rate of patients with prediabetes to normal glycemia, compared to conventional lifestyle intervention only.

Approval of protocol and consent forms by the local institutional review board was obtained at Peking University Health Science Center.

### 2.2. Trial Population

Individuals with high risk for diabetes were screened to confirm the glycemic state by oral glucose tolerance test (OGTT). High risk population included individuals with previously elevated fasting glucose level between 6.1 and 7.0 mmol/L or elevated 2-hour postprandial glucose level between 7.8 and 11.1 mmol/L. Our goal was to recruit 2000 participants form outpatient departments at 36 public hospitals in Beijing, China. Patients with prediabetes (confirmed by OGTT) were eligible for inclusion. Major inclusion criteria are listed as follows. 

*BPRP Study Major Inclusion Criteria*

- Voluntarily participating in the trial and signing subject's informed consent form
- Prediabetic patients
- Both males and females
- Not limited to ethnicity
- 25 years of age–70 years of age
- 22 kg/m^2^ ≤ BMI < 35 kg/m^2^

Meanwhile, detailed inclusion and exclusion criteria are listed in Supplementary Table 1 (see Supplementary Material available online at https://doi.org/10.1155/2017/7602408). Informed consent was obtained before the individuals could participate in any screening procedures. Eligible participants were then randomized into one of the four arms of the study.

### 2.3. Randomization and Follow-Up

Randomization was undertaken by an independent statistician using a computer generated random sequence and was performed as block randomization with a 1 : 1 : 1 : 1 allocation ratio in four arms. Sealed envelopes were used for random allocations at the study sites. Both the participants and healthcare providers were blinded by the medication, while they were open to the lifestyle intervention.

**Figure 1:**
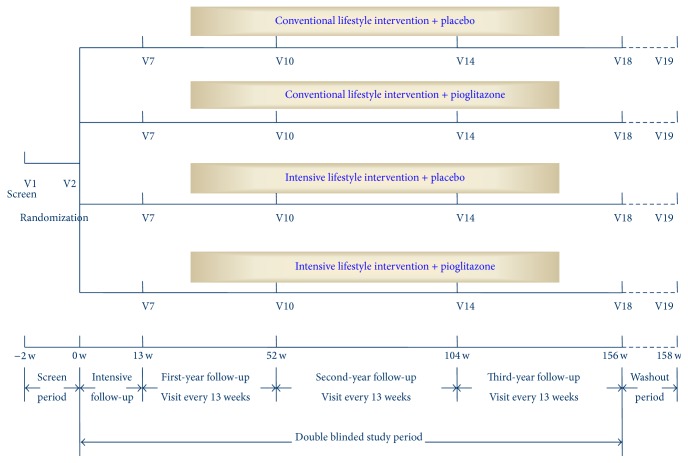
Study flow chart.

All the participants were recruited and followed up in the outpatient clinic in 36 study cites in Beijing. The follow-up of the study would last for three years with 19 scheduled visits. All participants would have an annual examination for glucose status (OGTT) during the follow-up period. Once the participants have been reverted to normal glucose state or have developed diabetes, defined by OGTT performed at annual examination, he or she would be terminated from the study (Figure 1).

For all four arms, study visits are scheduled for every 2-3 weeks during the first 13 weeks and every 13 weeks thereafter. Participants were required to complete a lifestyle diary comprising 3-day food records and average frequency of exercise per week for each visit.

In intensive lifestyle intervention group, participants would be educated at each visit after randomization, and the investigators would prescribe an individualized lifestyle prescription for them at each visit according to their body weight and lifestyle diary. A software program was developed to collect the lifestyle information from participants' lifestyle diary. Based on this information and the body weight at each visit, the software could calculate the compliance with lifestyle recommendations of each participant. The software would then generate a lifestyle prescription including the diet and exercise recommendations. Specific indicators of intensive lifestyle intervention are presented as follows. 

*Specific Indicators of Intensive Lifestyle Intervention*

*Exercise Principle*

- Mainly whole-body aerobic endurance exercise, moderate intensity (3–6 MET), ≥30 min/day, 3–7 days/week, ≥150 min/week, and 180–300 min/week, was recommended.
- Resistance exercise (resisted movement) as supplement was as follows: 40–50% of 1 repetition maximum (40–50% 1 RM), 3 sets of 8–10 exercises with 10–15 repetitions/set, and 2-3 days/week.
- Moderate stretching exercise and flexibility exercise were as follows: ≥15 min/day and 3–7 days/week.
- Energy consumption of exercise was as follows: total accumulative energy consumption ≥150 kcal/day, typically 150–300 kcal/day, and ≥750 kcal/week, typically 900–1500 kcal/week.
- Exercise was performed according to three stages, that is, adaption stage, consolidation stage, and maintenance stage.

*Diet Principle*

- Based on Harris-Benedict formula [16], according to the participant's specific condition, required calorie was calculated, and rational diet plan was made for the participant.

*The Goal of Weight Control*

- For those with BMI ≥ 24 (kg/m^2^), waist circumference ≥80 cm in females, or waist circumference ≥ 85 cm in males, weight should be reduced according to negative energy balance principle.
- The goal of weight loss was 5–10% of the current weight.
- Weight loss rate was 2–4 kg/month.

In conventional lifestyle intervention group, participants would receive the usual lifestyle modification advice at baseline and at annual visits, without any individualized counseling. They would not get a lifestyle evaluation and lifestyle related prescription. To avoid the contamination between groups, similar visit schedule was designed for all the groups.

Among participants who received pioglitazone, the dose of the medication (30 mg/day) remains the same throughout the follow-up period. The active pioglitazone and the matched placebo were manufactured by Beijing Taiyang Pharmaceutical Company. The supply chain of active medication and placebo was managed by the study investigators at the participating study centers.

Participants who were identified to have achieved normal glucose level at annual visit were asked to stop the medication and were invited for OGTT two weeks after the last visit. This procedure was followed for those participants who remained prediabetic during the course of 3 years of follow-up. Those who remained prediabetic or regressed back to normal glucose status were advised to seek usual care. Those who were found to have developed diabetes after 2 weeks of washout period were also advised to seek standard care for diabetes. All participants were advised to follow standard lifestyle management at the end of follow-up.

### 2.4. Primary and Secondary Outcomes

The primary aim of the study was to evaluate the proportions of participants regressing back to normal glucose level during follow-up. The normal glucose level was defined as FPG < 6.1 mmol/L and 2 hPG < 7.8 mmol/L during the OGTT. The secondary outcomes of the study were as follows: (1) incidence of type 2 diabetes, (2) time to achieving normal glucose level, (3) change in HbA1c, (4) change in body weight and waist circumference, (5) changes in blood pressure, LDL-cholesterol, HDL-cholesterol, and triglyceride, (6) changes in adiponectin, hsCRP, and insulin and C-peptide at fasting and after challenge, (7) change in urine albumin-creatinine ratio and serum creatinine, (8) composite of the incidence of at least one of the events—heart failure, nonfatal myocardial infarction, nonfatal stroke, or all-cause mortality, and (9) quality of life.

### 2.5. Study Measures

Details of study measurements are presented in Supplementary Table 2. At randomization and annual examinations, glucose tolerance status would be assessed by 75 g OGTT. OGTT was performed in the morning. All laboratory analyses are being conducted at the Peking University Peoples Hospital's central laboratory. Data on physical activity and diet habits would be collected from patients' diary. All lifestyle data are fed into the software to calculate the total calorie intake and physical activity level. HbA1c was measured by HPLC (Ultra2 HbA1c Detector; Primus Corporation, Duluth, GA, USA; normal range 4–6%, 20–42 mmol/mol). An immune-nephelometry method was used to measure the levels of LDL-cholesterol, HDL-cholesterol, and triacylglycerol (COBAS Integra 400 Plus System; Roche Diagnostics, Basel, Switzerland). Insulin and C-peptide were measured by an electrochemiluminescence immunoassay (Elecsys 2010 system; Roche Diagnostics). All the study drugs were withheld in the morning of testing.

At 13 weeks from randomization, ALT, AST, and serum creatinine would be measured to monitor side effects of pioglitazone and to rule out the participants who have had serious conditions which may not be suitable for the continuation of the study. Vital signs, body weight, waist circumference, and blood pressure would be recorded at each study visit. Urine HCG would also be tested at each study visit in order to avoid the use of pioglitazone during unexpected pregnancy in women within gestational age. Participants who were found pregnant during follow-up were terminated from the study.

### 2.6. Statistical Considerations

#### 2.6.1. Power Analysis

BPRP would recruit 2000 participants (500 in each arm) and would be followed for a planned maximum follow-up of 3 years. This sample size is expected to provide approximately 90% power with 5% type 1 error to detect 10% relative increase in the rate of primary outcomes among participants assigned to intensive lifestyle intervention compared with conventional lifestyle intervention group under the following assumptions:

1. 35.3% for the conventional lifestyle plus placebo, 44.3% for the conventional lifestyle plus pioglitazone, 45.3% for the intensive lifestyle plus placebo, and 54.3% for the intensive lifestyle plus pioglitazone.
2. Participants would be recruited in half a year.
3. 30% of the participants might be lost to follow-up during the whole study.

#### 2.6.2. Analysis Approach

The primary and secondary outcomes of the study will be evaluated following the intention-to-treat approach, with additional supporting analyses based on the per-protocol population. A separate Statistical Analysis Plan is in place which details the analysis approaches.

#### 2.6.3. Statistical Methods for Baseline Data Analysis

The study participants were randomized at baseline into four groups. However, the distributions of baseline study parameters are presented by the intensive and conventional lifestyle group and by different categories of age at randomization. Basic statistics were presented by number (%), mean (SD), or median (IQR) as appropriate. To evaluate the patterns of the distributions of glycemic parameters and body mass index by age groups, density plots were created. The distributions of the study parameters were not compared between the groups for possible differences.

## 3. Results

### 3.1. Patient Recruitment

From March 2007 to March 2011, 4397 individuals were screened who met the screening criteria. Among these individuals 2034 (46.3%) were identified to have prediabetes. Following the inclusion and exclusion criteria, 1954 eligible patients were randomized to four groups in equal proportion in 36 participating study centers.

### 3.2. Baseline Characteristics

In the study cohort, 42% were male, with mean (SD) age 53 (10) years, median (IQR) BMI 26.0 (23.9, 28.2) kg/m^2^, 49% were overweight and 12% were obese and 23% were current or ex-smokers (Table 1). Older patients were significantly less likely to be current or ex-smokers and obese, compared to patients below the age of 40 years (Table 2). Of all the participants, only 24% had low level physical activity.

**Figure 2:**
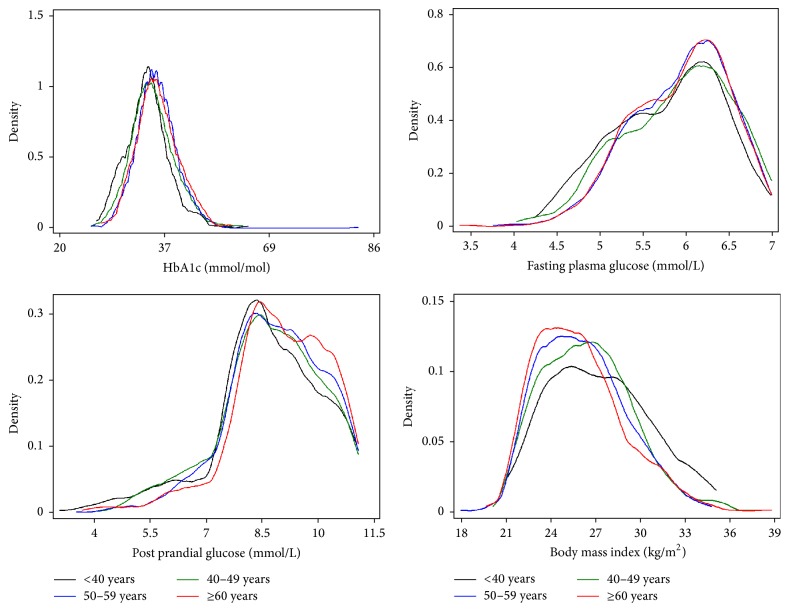
Density plots of HbA1c, fasting plasma glucose, postprandial glucose, and body mass index by age categories at randomization.

The distributions of fasting and postprandial plasma glucose levels were similar between lifestyle intervention groups and across the age groups at randomization (Tables 1 and 2, Figure 2). With an average HbA1c level of 5.3% (34 mmol/mol) at baseline, about 7% patients had HbA1c ≥ 6.5% (48 mmol/mol). About 15% participants were identified with isolated IFG, while most of the subjects (85%) had IGT (54% had isolated IGT and 31% had IFG plus IGT). The distribution of metabolic and other risk factors were similar across age groups.

## 4. Discussion

BPRP is the first study to determine whether lifestyle modification and/or pioglitazone could revert prediabetic state back to normoglycemia in Chinese population and to explore the mechanism through which different interventions exert their effects on glucose metabolism and cardiovascular risk factors. Compared with previous diabetes prevention studies, our study has several unique features. First, while most of the earlier studies evaluated the efficacy of different interventions to prevent the development of diabetes in individuals with IGT, our study aims at evaluating the efficacy of intensive lifestyle intervention with or without TZD to regress back the prediabetic individuals to normoglycemic status [5, 10, 13, 20–23]. Only few studies have examined the effect of intervention(s) on conversion into normoglycemia in individuals with prediabetes [12]. However, the regression back to normoglycemia was not the primary outcome of these studies.

In individuals with IGT, previous studies have showed that intensive lifestyle intervention can reduce the incidence of diabetes by 31%–58% [5, 15, 24]. The goal of lifestyle intervention, however, is difficult to achieve and maintain. Treatment of IGT with oral antidiabetic drugs, such as metformin, acarbose, or TZDs, has been shown to prevent or delay progression to diabetes in high risk individuals [10–12, 15] or prior gestational diabetes mellitus [25]. In addition, TZDs have shown greater efficacy in preventing IGT developing to diabetes, compared to that observed with acarbose or metformin. In IGT individuals receiving TZDs, the relative risk was reduced by 55–72% [11, 12, 25], compared to a risk reduction of 31% and 25% in IGT individuals receiving metformin [15] and acarbose [10], respectively.

Individuals with prediabetes receiving rosiglitazone were more likely to regress to normoglycemia compared with individuals receiving placebo [11]. After 5.7 years of median follow-up in DPP study, individuals who returned to normoglycemia at least once had a reduced risk of developing diabetes compared with individuals who consistently had prediabetes [26]. Increased *β*-cell function and insulin sensitivity may contribute to the reduced risk for diabetes in individuals who returned to normoglycemia during the intervention [26]. This suggests individuals who returned to normoglycemia may benefit more in terms of preventing diabetes. Studies aimed at evaluating the effect of intervention on conversion into normoglycemia in individuals with prediabetes and exploring the possible mechanisms involved in the conversion are needed.

Secondly, our prediabetic study population included both isolated elevated IFG and IGT population. Most of the earlier studies, including Da Qing study, DPP study, DPS study and ACT NOW trial, evaluated only the IGT population [5, 20, 21, 23]. The mechanism of isolated elevated fasting glucose level may be different with that of elevated postprandial glucose level. Only DREAM trial included individuals with IGT and with isolated IFG [22]. However, the primary outcome of this study was the incidence of diabetes during follow-up, and the efficacy of intensive lifestyle intervention was not evaluated with the intervention therapy (rosiglitazone and/or ramipril). Also, there is no data in Chinese population with isolated IFG. Our study would provide new insight into the possible efficacy of combination of lifestyle intervention and TZD in individuals with isolated IFG.

Thirdly, with a baseline BMI of 26 kg/m^2^, our study offered an excellent opportunity to evaluate the possible efficacy of intensive lifestyle intervention with or without intervention with ADD in normal weight and overweight individuals. The Da Qing study and the Indian Prevention Program [5, 13] had similar BMI in the study population. However, the primary outcomes and the interventions in these studies were different. Other studies, primarily based on Caucasians from Europe and America, show a higher BMI level at baseline (around 30 kg/m^2^) [12, 20, 22, 23]. However, our participants were not that obese as Caucasians in most previous diabetes prevention studies. So the goal we have set for intensive lifestyle intervention group might be a little bit difficult to achieve. But this may also provide us with an opportunity to find a proper goal of lifestyle intervention among normal weight population with prediabetes in the future.

Lastly, as there has been rapid development in the Chinese society and its lifestyle over the last decade, the general population is receiving more and more information from media on how to prevent diabetes. Therefore, in such an era of information explosion, it remains unknown whether individuals receiving intensive lifestyle intervention would at all show any significant benefit over those in the control group. Our study would provide necessary information to answer this question. To maximize the potential benefits from intensive lifestyle intervention, individualized lifestyle education and computerized prescription would be given to the participants in intensive lifestyle treatment group. This individualized education is based on the characteristics of each patient, considered with their body weight, diet habit, and exercise preference.

In summary, BPRP addresses the dramatically increasing population of prediabetes in China which is a major public health problem. A possible positive effect of the intensive lifestyle intervention on conversion rate from prediabetes into normoglycemia would provide a simple and powerful public health message. On the other hand, a finding that this intervention had no effect or was detrimental would be equally important and would indicate that efforts to improve diabetes care should be directed elsewhere.

## 5. Conclusion

BPRP was the first study to determine if lifestyle modification and/or pioglitazone could revert prediabetic state to normoglycemia in Chinese population. Major baseline parameters were balanced between two lifestyle intervention groups. In addition, with a baseline BMI of 26 kg/m^2^, our study also offers an excellent opportunity to evaluate the possible efficacy of intensive lifestyle intervention with or without intervention with antidiabetic drug in normal weight and overweight individuals. Our study addresses the dramatically increasing population of prediabetes in China which is a major public health problem. This randomized clinical trial would provide the evidence of whether intensive lifestyle intervention and/or pioglitazone might convert prediabetes back into normoglycemia and would also quantify the benefits of the conversion into normoglycemia in different intervention groups.

## Supplementary Material

The supplementary materials inculde the detailed inclusion and exclusion criteria of the trial, as well as the study scedule of both the intensive lifestyle intervention group and the conventional lifestyle intervention group.

## Figures and Tables

**Table 1:** Characteristics of participants by different lifestyle intervention at randomization.

	Conventional	Intensive	All
*N*	972 (50)	973 (50)	1945
Sex			
Male	450 (46)	374 (38)	824 (42)
Age (years)			
mean ± SD	52 ± 10	53 ± 10	53 ± 10
Occupation			
Professional/business	447 (47)	427 (45)	874 (46)
Workers	59 (6)	53 (6)	112 (6)
Retired	362 (38)	396 (42)	758 (40)
Jobless/other	83 (9)	76 (8)	159 (8)
Ethnicity			
Han	934 (97)	927 (96)	1861 (96)
Others	31 (3)	41 (4)	72 (4)
Education level			
Low	46 (5)	42 (4)	88 (5)
Middle	468 (49)	449 (47)	917 (48)
High	436 (46)	468 (49)	904 (47)
Smoking state			
Current smoker or past smoker	234 (24)	202 (21)	436 (23)
Body shape			
BMI (kg/m^2^)	26 (24, 28)	26 (24, 28)	26 (24, 28)
Normal: BMI < 25	385 (40)	360 (37)	745 (38)
Overweight: 25 ≤ BMI < 30	464 (48)	495 (51)	959 (49)
Obese: BMI ≥ 30	123 (13)	118 (12)	241 (12)
Waist (cm)	89 (83, 96)	88 (82, 95)	89 (82, 95)
Blood pressure			
Systolic blood pressure (mmHg)	120 (111, 130)	120 (110, 130)	120 (110, 130)
Diastolic blood pressure (mmHg)	79 (70, 81)	77 (70, 80)	78 (70, 80)
Glucose level			
Fasting plasma glucose (mmol/L)	6.0 (5.5, 6.4)	6.0 (5.5, 6.4)	6.0 (5.5, 6.4)
2h plasma glucose (mmol/L)	8.8 (8.1, 9.8)	8.9 (8.1, 9.9)	8.9 (8.1, 9.9)
HbA1c (%)	5.8 (5.6, 6.0)	5.8 (5.5, 6.1)	5.8 (5.6, 6.1)
HbA1c (mmol/mol)	40 (38, 42)	40 (37, 43)	40 (38, 43)
HbA1c < 5.7% (39 mmol/mol)	331 (34)	327 (34)	658 (34)
5.7% (39 mmol/mol) ≤ HbA1c < 6.5% (48 mmol/mol)	572 (59)	572 (59)	1144 (59)
HbA1c ≥ 6.5% (48 mmol/mol)	69 (7)	74 (8)	143 (7)
IGT	821 (85)	833 (86)	1654 (85)
Isolated IGT	532 (55)	525 (54)	1057 (54)
IFG + IGT	289 (30)	308 (32)	597 (31)
Isolated IFG	151 (16)	140 (14)	291 (15)
Lipid level			
Total cholesterol (mmol/L)	4.9 (4.3, 5.5)	4.9 (4.3, 5.4)	4.9 (4.3, 5.5)
LDL-C (mmol/L)	3.2 (2.7, 3.7)	3.1 (2.6, 3.7)	3.2 (2.6, 3.7)
HDL-C (mmol/L)	1.2 (1.0, 1.4)	1.2 (1.0, 1.4)	1.2 (1.0, 1.4)
Triglyceride (mmol/L)	1.5 (1.1, 2.1)	1.5 (1.1, 2.0)	1.5 (1.1, 2.1)
Liver function			
ALT (U/L)	21 (16, 31)	21 (15, 29)	21 (16, 30)
AST (U/L)	22 (18, 26)	21 (17, 26)	21 (18, 26)
Hemoglobin			
Hemoglobin (g/L)	143 (134, 153)	141 (132, 152)	142 (133, 153)
HOMA			
HOMA-IR	2.4 (1.6, 3.5)	2.4 (1.6, 3.6)	2.4 (1.6, 3.5)
HOMA-beta	77. 5 (50.8, 113.1)	79.9 (52.3, 116.7)	78.5 (51.8, 114.7)
Cytokines			
CRP (*μ*mol/L)	1.2 (0.7, 2.3)	1.2 (0.7, 2.4)	1.2 (0.7, 2.4)
Adiponectin	6.2 (4.2, 8.8)	6.2 (4.3, 9.0)	6.2 (4.3, 8.9)
SOD	6.8 (4.1, 10.3)	6.9 (4.3, 10.5)	6.9 (4.2, 10.4)
Amylin	7.7 (6.5, 9.5)	7.5 (6.4, 9.3)	7.6 (6.4, 9.4)
IL-6	2.3 (1.5, 4.2)	2.3 (1.5, 3.8)	2.3 (1.5, 4.0)
Urine ACR			
Urine albumin/Cr (mg/g)	7.4 (4.5, 15.2)	7.4 (4.4, 14.3)	7.4 (4.5, 14.7)
Diet			
Daily calories intake (kcal/d)	1521 (1242, 1874)	1554 (1248, 1920)	1535 (1243, 1899)
Proportion of total calories intake from carbohydrate (%)	60 (51, 69)	59 (50, 67)	60 (50, 68)
Proportion of total calories intake from protein (%)	14 (12, 16)	14 (12, 17)	14 (12, 17)
Proportion of total calories intake from fat (%)	23 (17, 30)	24 (18, 31)	24 (17, 30)
Physical activity			
Low level	232 (25)	224 (24)	456 (24)
Medium level	483 (52)	501 (53)	984 (53)
High level	223 (24)	213 (23)	436 (23)

*Note*. Estimates for continuous study parameters are presented by median (IQR), unless otherwise stated. Categorical study parameters are presented by number (percentage).

**Table 2:** Characteristics of participants by different age groups at randomization.

	<40 years old	40–49 y	50–59 y	≥60 y
Total				
*N*	237 (12)	462 (234)	802 (41)	444 (23)
Sex				
Male	138 (58)	238 (52)	272 (34)	176 (40)
Occupation				
Professional/business	185 (80)	330 (73)	313 (40)	46 (11)
Workers	17 (7)	35 (8)	46 (6)	14 (3)
Retired	0 (0)	37 (8)	367 (47)	354 (82)
Jobless/others	30 (13)	48 (11)	62 (8)	19 (4)
Ethnicity				
Han	226 (96)	446 (97)	775 (97)	414 (94)
Others	9 (4)	16 (4)	22 (3)	25 (6)
Education level				
Low	1 (0)	12 (3)	37 (5)	38 (9)
Middle	63 (27)	194 (43)	454 (58)	206 (48)
High	170 (73)	250 (55)	299 (38)	185 (43)
Smoking state				
Smoker (current or past)	76 (32)	143 (31)	145 (18)	72 (16)
Body shape				
BMI (kg/m^2^)	27 (25, 30)	26 (24, 28)	26 (24, 28)	25 (24, 28)
Normal: BMI < 25	73 (31)	164 (36)	316 (39)	192 (43)
Overweight: 25 ≤ BMI < 30	111 (47)	246 (53)	398 (50)	204 (46)
Obese: BMI ≥ 30	53 (22)	52 (11)	88 (11)	48 (11)
Waist (cm)	90 (84, 98)	90 (83, 96)	88 (82, 94)	88 (83, 95)
Blood pressure				
Systolic blood pressure (mmHg)	118 (110, 123)	120 (110, 126)	120 (112, 130)	125 (119, 133)
Diastolic blood pressure (mmHg)	76 (70, 80)	78 (70, 81)	79 (70, 81)	78 (70, 80)
Glucose level				
Fasting plasma glucose (mmol/L)	5.9 (5.3, 6.3)	6.0 (5.4, 6.4)	6.0 (5.5, 6.4)	6.0 (5.5, 6.3)
2 h plasma glucose (mmol/L)	8.7 (7.9, 9.6)	8.8 (8.0, 9.7)	9.0 (8.1, 9.9)	9.0 (8.3, 10.0)
HbA1c (%)	5.7 (5.4, 5.9)	5.7 (5.5, 6.0)	5.8 (5.6, 6.1)	5.8 (5.6, 6.1)
HbA1c (mmol/mol)	39 (36, 41)	39 (37, 42)	40 (38, 43)	40 (38, 43)
HbA1c < 5.7% (39 mmol/mol)	114 (48)	180 (39)	237 (30)	127 (29)
5.7% (39 mmol/mol) ≤ HbA1c < 6.5% (48 mmol/mol)	112 (47)	248 (54)	507 (63)	277 (62)
HbA1c ≥ 6.5% (48 mmol/mol)	11 (5)	34 (7)	58 (7)	40 (9)
IGT	198 (84)	379 (82)	680 (85)	397 (89)
Isolated IGT	142 (60)	253 (55)	425 (53)	237 (53)
IFG + IGT	56 (24)	126 (27)	255 (32)	160 (36)
Isolated IFG	39 (17)	83 (18)	122 (15)	47 (11)
Lipid level				
Total cholesterol (mmol/L)	4.6 (4.0, 5.2)	4.8 (4.2, 5.3)	5.0 (4.3, 5.7)	4.9 (4.3, 5.5)
LDL-C (mmol/L)	2.9 (2.5, 3.4)	3.1 (2.6, 3.6)	3.3 (2.7, 3.8)	3.2 (2.7, 3.7)
HDL-C (mmol/L)	1.1 (1.0, 1.3)	1.2 (1.0, 1.3)	1.2 (1.0, 1.4)	1.3 (1.1, 1.5)
Triglyceride (mmol/L)	1.7 (1.2, 2.5)	1.5 (1.1, 2.1)	1.5 (1.1, 2.1)	1.4 (1.1, 1.9)
Liver function				
ALT (U/L)	26 (18, 43)	22 (16, 32)	21 (16, 29)	18 (14, 24)
AST (U/L)	22 (18, 28)	20 (17, 26)	22 (18, 26)	21 (18, 25)
Hemoglobin				
Hemoglobin (g/L)	148 (135, 161)	146 (134, 157)	140 (133, 150)	140 (131, 148)
HOMA				
HOMA-IR	3.2 (1.8, 4.2)	2.5 (1.5, 3.4)	2.4 (1.7, 3.5)	2.3 (1.5, 3.3)
HOMA-beta	103.8 (66.0, 157.1)	80.1 (51.9, 115.8)	75.4 (51.5, 109.9)	73.4 (48.7, 104.1)
Cytokines				
CRP (*μ*mol/L)	1.1 (0.6, 2.3)	1.1 (0.7, 2.4)	1.2 (0.7, 2.4)	1.3 (0.7, 2.4)
Adiponectin	5.2 (3.7, 7.0)	5.6 (3.7, 8.2)	6.3 (4.6, 9.3)	6.9 (4.7, 9.5)
SOD	6.5 (3.7, 9.7)	7.0 (4.3, 10.4)	6.9 (4.1, 10.8)	6.7 (4.5, 10.0)
Amylin	7.7 (6.6, 9.4)	7.6 (6.5, 9.5)	7.5 (6.4, 9.3)	7.5 (6.3, 9.7)
IL-6	2.2 (1.5, 4.2)	2.0 (1.5, 3.4)	2.4 (1.5, 4.1)	2.5 (1.6, 4.3)
Urine ACR				
Urine albumin/Cr (mg/g)	6.4 (4.1, 13.5)	7.4 (4.3, 14.3)	7.4 (4.5, 14.5)	7.7 (4.8, 16.9)
Diet				
Daily calories intake (kcal/d)	1619 (1259, 2032)	1557 (1230, 1946)	1512 (1254, 1841)	1515 (1244, 1893)
Proportion of total calories intake from carbohydrate (%)	58 (50, 66)	59 (49, 68)	59 (50, 68)	61 (53, 68)
Proportion of total calories intake from protein (%)	14 (12, 17)	15 (12, 17)	14 (12, 17)	14 (12, 16)
Proportion of total calories intake from fat (%)	25 (19, 31)	24 (17, 31)	24 (18, 30)	23 (16, 29)
Physical activity				
Low level	95 (42)	133 (30)	168 (22)	60 (14)
Medium level	108 (47)	241 (54)	411 (53)	224 (53)
High level	25 (11)	75 (17)	195 (25)	141 (33)

*Note*. Estimates for continuous study parameters are presented by median (IQR), unless otherwise stated. Categorical study parameters are presented by number (percentage).

## Abbreviations

ACR: Albumin/creatinine
ADD: Antidiabetes drug
BMI: Body mass index
BPRP: Beijing Prediabetes Reversion Program
CRP: C-reactive protein
CVD: Cardiovascular disease
DPP: Diabetes prevention program
DPS: Finnish diabetes prevention study
HbA1c: Hemoglobin A1c
HDL-C: HDL-cholesterol
HOMA-beta: Homeostatic model assessment for beta cell function
HOMA-IR: Homeostatic model assessment for insulin resistance
IFG: Impaired fasting glucose
IGT: Impaired glucose tolerance
IL-6: Interleukin-6
LDL-C: LDL-cholesterol
OGTT: Oral glucose tolerance test
PG: Plasma glucose
SOD: Superoxide dismutase
TC: Total cholesterol
TG: Triglyceride
TZD: Thiazolidinedione.
