# Supplementary material for: Rationale, Design, and Baseline Characteristics of Beijing Prediabetes Reversion Program: A Randomized Controlled Clinical Trial to Evaluate the Efficacy of Lifestyle Intervention and/or Pioglitazone in Reversion to Normal Glucose Tolerance in Prediabetes
Source: J Diabetes Res. 2017 Jan 12;2017:7602408. doi: 10.1155/2017/7602408 (PMC5266841; doi:10.1155/2017/7602408)
Supplement: Supplementary file 1 — The supplementary materials inculde the detailed inclusion and exclusion criteria of the trial, as well as the study scedule of both the intensive lifestyle intervention group and the conventional lifestyle intervention group. [file 7602408.f1.pdf]

Supplementary Table 1. BPRP study inclusion and exclusion criteria

Inclusion Criteria

- Voluntarily participate the trial and sign subject's informed consent form
- Pre-diabetic patients

  Definition of pre-diabetes:
  - Impaired fasting glucose (IFG): FPG≥6.1mmol/L (110mg/dl) and <7.0mmol/L (126mg/dl), meanwhile 2hPG<7.8mmol/L (140mg/dl) after 75g oral glucose load;
  - Impaired glucose tolerance (IGT): FPG<7.0mmol/L (126mg/dl), meanwhile, 2hPG ≥7.8mmol/L (140mg/dl) and <11.1mmol/L (200mg/dl) after 75g oral glucose load.
  - According to WHO diabetes classification standard, if meeting both IGT and IFG criteria, the patient would be diagnosed with IGT.
- Both males and females.
- Not limited to ethnicity.
- 25 years of age - 70 years of age.
- 22kg/m2≤BMI＜35 kg/m2。

Exclusion Criteria

- The patient can be diagnosed with diabetes at baseline, meeting any of the following criteria:
  - Fasting plasma glucose ≥7.0 mmol/L (126 mg/dL);
  - The 2 hour OGTT plasma glucose level ≥11.1 mmol/L (200 mg/dL);
  - Previous diagnosed diabetes, confirmed by clinical data (such as fasting plasma glucose≥7.0 mmol/L [126 mg/dL] or diabetes diagnosed by OGTT). In those who reports previous diagnosis of diabetes, without using hypoglycemic agents or clinical data confirming diabetes, OGTT should be performed to determine the glucose tolerance state;
  - Previous use of hypoglycemic agents (except gestational diabetes).
- Newly diagnosed coronary heart disease within 6 months, newly occurred myocardial infarction or unstable angina within 6 months, or hospitalization for heart disease or stroke within 6 months.
- New York Heart Association Class III or IV congestive heart failure, or current usage of loop diuretic or digitaloid drugs.
- Left bundle branch block, III degree atrioventricular block, or other severe arrhythmia.
- Uncontrolled hypertension: systolic pressure > 160 mmHg or diastolic pressure > 100 mmHg on any blood pressure measurement with medication. For mild hypertension, it is advised to receive preliminary observation and treatment, and those patients should not be excluded.
- Other severe cardiovascular and cerebrovascular diseases that clinician considers not suitable for participation in this study.
- Severe pulmonary diseases.
- Recent abdominal surgery or past history of major abdominal surgery.

- Gastrointestinal diseases (pancreatitis, inflammatory bowl disease).
- The patient reports chronic hepatitis or liver cirrhosis, or increase of serum ALT or AST level, meeting the following standard: serum ALT ⩾ 2.5 folds of upper limit of normal range or serum AST ⩾ 2.5 folds of upper limit of normal range.
- With any of the following urological diseases:
  - Serum creatinine ⩾133 μmol/L (1.5 mg/dL);
  - Positive quantitative determination of urine protein without urological infection or contamination with vaginal discharge.
  - Past history of bladder cancer.
  - Gross hematuria or microscopic hematuria (red blood cell > 3 per high power field)
- In blood routine test, hemoglobin < 110 g/L.
- Current diagnosis of Grave's disease or hypothyroidism regardless of the current thyroid function.
- Current diagnosis endocrine diseases blocking glucose metabolism (Cushing's syndrome, acromegaly, pheochromocytoma, prolactinoma and hypoglycemia).
- Fasting plasma triglyceride level > 6.77 mmol/L (600 mg/dL).
- Cancer regardless the requirement of treatment.
- Current diagnosis of severe physical functional diseases that may limit the physical activity.
- Current diagnosis of tuberculosis or is using anti-tuberculosis drugs.
- With any of the following situations: severe psychological disorders, is using antipsychotics, alcohol abuse or dependence, drug abuse or dependence.
- Current use of any following drugs: thiazide diuretics, β receptor blockers, nicotinic acid drugs to lower lipid, systemic glucocorticoids (not including topical, eye and inhalation application), weight-reducing medication (including use within the past 3 months).
- Allergic to pioglitazone hydrochloride or its component.
- Pregnant or nursing women (not including women who will terminate nursing within 6 weeks); Woman who is planning to be pregnant during the study, and/or who can not take effective contraception measures before enrollment in the trial and/or during the trial (effective contraception measures include sterilization, intrauterine contraceptive device, oral contraceptives and condom).
- In past 6 months, weight loss exceeding 10% of the total original body weight caused by any reason except exercise.
- Another member of the household is a participant or staff member in BPRP.
- Current participation in other clinical trial at present or within the past 3 months.
- Travel plans that do not permit full participation.
- Other medical, psychiatric, or behavioral limitations that in the judgment of the principal investigator may interfere with study participation or the ability to follow the intervention protocol.

Supplementary Table 2. Study schedule

Visit	V1§	V2	V3	V4	V5	V6	V7
Time relative to V2 (week)	-2	0	2	4	7	10	13
Visit window (±day)		7	3	3	7	7	7
Informed Consent	X						
Medical history and concomitant disease	X						
Inclusion and exclusion criteria	X						
Concomitant medication	X	X	X	X	X	X	X
Adverse event		X	X	X	X	X	X
Physical examination	X						
Body measurement (waist circumference, hip circumference, body height)	X						
Vital signs and body weight	X			X	X	X	X
Baseline survey and assessment of nutrition and exercise		X					
Follow-up survey and assessment of nutrition and exercise				X	X	X	X
Health questionnaire		X					
Health education			X	Intensive [1]	Intensive	Intensive	Intensive
ECG (12-lead) [2]	X						
OGTT (FPG*, 2hPG*, fasting insulin△, C peptide△)	X						
Blood and urine routine*	X						
Urine albumin/creatinine ratio△		X					
Urine HCG [3] *	X			X	X	X	X
ALT, AST, CREA*	X						X [4]
TG*	X						
T-CHO, LDL-C, HDL-C, TG HbA1c, hsCRP, adiponectin△		X					
Random grouping, distribute pedometer		X					
Distribute drugs and patient diary		X	X	X	X	X	X
Return drugs and patient diary			X	X	X	X	X
Make an appointment for examination	X	X	X	X	X	X	X

[1] Refers to intensive group.

[2] ECG performed between 4 weeks prior to V1 and the day of V1 can be used as V1 data.

[3] This test is only for women of child-bearing age (including menopause for less than 1 year). The test must be performed in V1 and V18. In other visits, the test is only performed for women of child-bearing age (including menopause for less than 1 year) whose menstruation has been delayed for 10 days or more.

[4] Only ALT and AST are tested.

* Examination is performed in a local laboratory.

▵ Examination is performed in the central laboratory.

§ For women of child-bearing age, V1, V10, V14 and V18 visits should be arranged during non-menstrual period. If these visits occur during menstrual period, urine routine and urine HCG (V1 and V18) tests should be performed within 3 days after the end of menstruation.

Visit	V8	V9	V10§	V11	V12	V13	V14§
Time relative to V2 (week)	26	39	52	65	78	91	104
Visit window (±day)	14	14	14	14	14	14	14
Informed Consent							
Medical history and concomitant disease							
Inclusion and exclusion criteria, re-screening criteria							
Concomitant medication	X	X	X	X	X	X	X
Adverse event	X	X	X	X	X	X	X
Physical examination			X				X
Body measurement (waist circumference, hip circumference, body height)			X				X
Vital signs and body weight	X	X	X	X	X	X	X
Baseline survey and assessment of nutrition and exercise							
Follow-up survey and assessment of nutrition and exercise	X	X	X	X	X	X	X
Health questionnaire			X				
Health education	Intensive [1]	Intensive	X	Intensive	Intensive	Intensive	X
ECG (12-lead) [2]			X				X
OGTT (FPG*, 2hPG*, fasting insulin△, C peptide△)			X				X
Blood and urine routine*			X				X
Urine albumin/creatinine ratio△			X				X
Urine HCG [3] *	X	X	X	X	X	X	X
ALT, AST, CREA*			X				X
TG*							

Visit	V8	V9	V10§	V11	V12	V13	V14§
Time relative to V2 (week)	26	39	52	65	78	91	104
T-CHO, LDL-C, HDL-C, TG HbA1c, hsCRP, adiponectin△			X				X
Random grouping, distribute pedometer							
Distribute drugs and patient diary	X	X	X	X	X	X	X
Return drugs and patient diary	X	X	X	X	X	X	X
Make an appointment for examination	X	X	X	X	X	X	X

[1] Refers to intensive group.

[2] ECG performed between 4 weeks prior to V1 and the day of V1 can be used as V1 data.

[3] This test is only for women of child-bearing age (including menopause for less than 1 year). The test must be performed in V1 and V18. In other visits, the test is only performed for women of child-bearing age (including menopause for less than 1 year) whose menstruation has been delayed for 10 days or more.

[4] Only ALT and AST are tested.

* Examination is performed in a local laboratory.

△ Examination is performed in the central laboratory.

§ For women of child-bearing age, V1, V10, V14 and V18 visits should be arranged during non-menstrual period. If these visits occur during menstrual period, urine routine and urine HCG (V1 and V18) tests should be performed within 3 days after the end of menstruation.

Visit	V15	V16	V17	V18§	V19
Time relative to V2	117	130	143	156	V18 + 2 weeks◆
Visit window (±day)	14	14	14	14	3
Informed Consent					
Medical history and concomitant disease					
Inclusion and exclusion criteria, re-screening criteria					
Concomitant medication	X	X	X	X	X
Adverse event	X	X	X	X	X
Physical examination				X	
Body measurement (waist circumference, hip circumference, body height)				X	
Vital signs and body weight	X	X	X	X	
Baseline survey and assessment of nutrition and exercise					
Follow-up survey and assessment of nutrition and exercise	X	X	X	X	
Health questionnaire				X	
Health education	Intensive [1]	Intensive	Intensive	X	
ECG (12-lead) [2]				X	
OGTT (FPG*, 2hPG*, fasting insulin△, C peptide△)				X	X [5]
Blood and urine routine*				X	
Urine albumin/creatinine ratio△				X	
Urine HCG [3] *	X	X	X	X	
ALT, AST, CREA*				X	
TG*					
T-CHO, LDL-C, HDL-C, TG<br>HbA1c, hsCRP, adiponectin△				X	
Random grouping, distribute pedometer					
Distribute drugs and patient diary	X	X	X		
Return drugs and patient diary	X	X	X	X	

Visit	V15	V16	V17	V18§	V19
**Time relative to V2**	**117**	**130**	**143**	**156**	**V18 + 2 weeks◆**
**Make an appointment for examination**	**X**	**X**	**X**	**X**	

[1] Refers to intensive group.

[2] ECG performed between 4 weeks prior to V1 and the day of V1 can be used as V1 data.

[3] This test is only for women of child-bearing age (including menopause for less than 1 year). The test must be performed in V1 and V18. In other visits, the test is only performed for women of child-bearing age (including menopause for less than 1 year) whose menstruation has been delayed for 10 days or more.

[5] Only FPG and 2hPG are tested. This is only performed for patients who are not diagnosed with diabetes in V18 OGTT.

* Examination is performed in a local laboratory.

△ Examination is performed in the central laboratory.

§ For women of child-bearing age, V1, V10, V14 and V18 visits should be arranged during non-menstrual period. If these visits occur during menstrual period, urine routine and urine HCG (V1 and V18) tests should be performed within 3 days after the end of menstruation.

◆ Date of V19 is 2 weeks ± 3 days relative to date of V18.
